# Editorial: Genomic and transcriptomic insights into ESKAPE pathogens' antimicrobial resistance

**DOI:** 10.3389/fmicb.2026.1999152

**Published:** 2026-09-29

**Authors:** Taru Singh, Sajad Ahmad Dar, Sayim Wani, Shukla Das

**Affiliations:** 1 Amity Institute of Biotechnology, Amity University, Noida, India; 2 Department of Nursing, College of Nursing and Health Sciences, Jazan University, Jazan, Saudi Arabia; 3 Department of Microbiology, Maharishi Markandeshwar Institute of Medical Sciences and Research (MMIMSR), Ambala, India; 4 Department of Microbiology, University College of Medical Sciences (University of Delhi) and Guru Teg Bahadur Hospital, Delhi, India

**Keywords:** antimicrobial resistance, ESKAPE pathogens, evolution, genomic surveillance, transcriptomics

Antimicrobial resistance (AMR) is sustained by the extraordinary capacity of bacterial pathogens to acquire mobile resistance determinants, remodel gene expression, and adapt under antimicrobial and host-imposed pressures. These processes are especially consequential among the ESKAPE pathogens—*Enterococcus faecium, Staphylococcus aureus, Klebsiella pneumoniae, Acinetobacter baumannii, Pseudomonas aeruginosa*, and *Enterobacter* spp., and the frequently included *Escherichia coli* (collectively termed ESKAPEE), which together account for a substantial burden of difficult-to-treat infections. Their success reflects more than resistance alone: many lineages persist in healthcare environments, form biofilms, colonize vulnerable hosts, exchange mobile genetic elements, and combine resistance with enhanced virulence. This combination complicates empirical therapy, infection prevention, and the preservation of last-resort antimicrobials.

Genomic and transcriptomic approaches now allow AMR to be examined not simply as the presence of a resistance gene, but as a dynamic phenotype shaped by lineage, genome context, expression, protein structure, virulence, and within-host evolution. Whole-genome sequencing can locate resistance determinants within chromosomes, plasmids, integrons, and other mobile elements, while phylogenomics can identify transmission and dissemination patterns. Transcriptomics adds a functional layer by revealing regulatory responses under antimicrobial or environmental pressure. Neither approach replaces conventional susceptibility testing or experimental validation; their greatest value emerges when molecular observations are interpreted alongside phenotype, epidemiology, and clinical outcome.

Rapid diagnostics are most useful when their biological and clinical limits are understood. Sun et al. combined MALDI-TOF mass spectrometry with machine learning to rapidly predict vancomycin-resistant *E. faecium*. Their work shows the promise of routinely generated spectral data for earlier resistance classification, while the reduced performance of external-only models underscores the need for locally representative datasets and careful validation before clinical deployment. Complementing this data-driven approach, Diab et al. investigated *E. coli* isolates carrying OXA-48-like carbapenemase genes despite meropenem-susceptible phenotypes. Whole-genome analysis and structural modeling linked particular enzyme substitutions and genomic contexts to this discordance. Together, these studies caution against treating any single molecular signal as definitive: rapid prediction, resistance-gene detection, and phenotypic susceptibility testing provide different but complementary information.

Several contributions demonstrate that resistance can emerge through adaptation during antimicrobial exposure or prolonged infection. Using whole-genome sequencing and RNA sequencing, Syed et al. characterized cefiderocol resistance selected in clinical *E. coli* strains. Mutations affecting iron transport, membrane function, and metabolism, together with altered expression of iron-transport genes and small-colony-variant phenotypes, revealed multiple routes to resistance and associated fitness changes. This is particularly relevant for cefiderocol because bacterial iron-uptake systems contribute to its entry, creating several possible adaptive routes under drug pressure. Zhang F. et al. examined two related, mucoid, imipenem-resistant *P. aeruginosa* isolates recovered from one patient with silicosis. Mutations involving *mucA, oprD*, and *relA*, alongside differences in biofilm formation and stress responses, illustrated how persistence may reflect a balance between adaptive traits and fitness costs. These studies emphasize the value of longitudinal sampling and paired genomic–transcriptomic or genomic–phenotypic analysis for following bacterial evolution rather than viewing an infection through a single isolate. They also suggest that surveillance of newly introduced agents should include evolutionary experiments and serial clinical isolates, enabling recurrent adaptive pathways to be recognized before they become widespread.

The importance of linking genome content to experimentally measured pathogenicity is further illustrated by de Sales et al., Their comparative genomic analysis of *P. aeruginosa*, integrated with a *Caenorhabditis elegans* infection model, found that epidemic success was not synonymous with uniformly greater acute virulence. Instead, mobile resistance determinants, biofilm capacity, and accessory-genome features helped distinguish the persistence and dissemination of high-risk clones. This finding argues for genomic reports that go beyond clone labels and gene counts to include the genomic context and functional consequences of identified determinants.

Three studies focus on the convergence of resistance, virulence, and genomic plasticity in *K. pneumoniae*. Bezdicek et al. placed K1/ST23 isolates from the Czech Republic in a global phylogenomic context. Their identification of plasmid restructuring, chromosomal integration of virulence loci, and virulence–resistance fusion plasmids highlights routes by which hypervirulent lineages may progress toward multidrug resistance. Zhu et al. characterized polymyxin-resistant clinical isolates and found that chromosomal regulatory changes, particularly those affecting two-component systems, were more prominent than plasmid-mediated *mcr* genes. The presence of virulent phenotypes in a subset of these multidrug-resistant isolates reinforces the threat posed by resistance to last-resort drugs. From a different evolutionary perspective, Dai et al. used genomic, proteomic, transcriptomic, metagenomic, and phenotypic analyses to characterize an attenuated mutant derived from a hypervirulent strain during phage-isolation work. Its structural variation, altered morphology, reduced biofilm formation, and loss of virulence demonstrate how extensive genomic change can reshape pathogen fitness and host interaction. Collectively, these studies show why surveillance must track resistance and virulence together and why multi-omics findings require functional confirmation.

Clinical and regional surveillance provides the epidemiological framework in which molecular findings acquire practical meaning. In a 5-year study from an intensive care unit in Mogadishu, Ibrahim et al. documented a high burden of multidrug resistance among ESKAPEE isolates, including substantial carbapenem resistance in *A. baumannii* and *K. pneumoniae*. The study supplies important baseline evidence from a setting in which longitudinal AMR data remain limited and can inform empirical treatment policies and stewardship priorities. Abubakari et al. mapped regional evidence from Ghana and identified marked geographical disparities, methodological heterogeneity, and surveillance blind spots across human, animal, and environmental settings. Their review also illustrates how uneven research coverage can distort national estimates and leave emerging resistance mechanisms undetected. These contributions show that global surveillance depends on strong, standardized local systems and that One Health strategies must account for regional variation rather than assume a uniform national resistance landscape. Expanding sequencing capacity is important, but comparable sampling strategies, quality-assured microbiology, contextual metadata, and sustained reporting networks are equally necessary if genomic surveillance is to guide action.

The clinical consequences of high-risk lineages are evident in the study by Zhang D. et al. of endogenous endophthalmitis in northeastern China. Whole-genome sequencing of *K. pneumoniae* isolates revealed the predominance of hypervirulent ST23-K1, while clinical analysis highlighted the importance of identifying systemic foci and providing timely intravitreal therapy. This work connects molecular epidemiology with patient outcomes and demonstrates the value of integrating genomic data with detailed clinical information.

Finally, addressing AMR requires therapeutic innovation as well as improved detection. Hu et al. described Scymicrosin7-26, a mud-crab-derived antimicrobial peptide with activity against multidrug-resistant pathogens, inhibition of biofilm formation, and anti-inflammatory effects. Its membrane-disruptive and DNA-binding activities, physiological-salt stability, and low toxicity in the tested *in vitro* systems support further preclinical evaluation. Nevertheless, translation will require rigorous assessment of pharmacokinetics, delivery, *in vivo* efficacy, safety, and the potential for resistance.

Taken together, this Research Topic shows that AMR cannot be adequately understood from gene detection alone. Genomics defines lineages, mobile elements, and evolutionary change; transcriptomics identifies condition-dependent responses; proteomics and structural analysis help explain functional consequences; and experimental models and clinical surveillance establish biological and practical relevance. The next phase of AMR research should therefore prioritize interoperable datasets, representative sampling, longitudinal designs, and prospective validation across laboratories and populations. By integrating molecular resolution with phenotypic testing, clinical context, One Health surveillance, and therapeutic development, the field can move from describing resistance after it appears toward recognizing its trajectories earlier and intervening more effectively.

